# Average Shear Rates in the Screw Elements of a Corotating Twin-Screw Extruder

**DOI:** 10.3390/polym13020304

**Published:** 2021-01-19

**Authors:** Bruno Vergnes

**Affiliations:** CEMEF, MINES ParisTech, PSL Research University, UMR CNRS 7635, 06904 Sophia Antipolis, France; bruno.vergnes@mines-paristech.fr

**Keywords:** twin-screw extruder, screw element, average shear rate

## Abstract

The rapid estimation of the average shear rate encountered by the material as it flows along the screw elements of a corotating twin-screw extruder is a key point for many applications. In this paper, two methods of evaluation are presented that allow the calculation of the average shear rate as a function of the screw geometry, feed rate, and screw speed. A comparison is made between the approximate and exact methods. It is shown that it is crucial to take into account the shear component due to the pressure flow, especially in the left-handed screw elements.

## 1. Introduction

Twin-screw extrusion is a widely used technique with many applications in the field of polymer processing, especially for mixing and compounding applications [1,2]. From an engineering point of view, it is important to be able to rapidly obtain an accurate estimate of the shear rates encountered during flow along the screw elements. Indeed, this allows the correct estimation of significant parameters of the process, such as viscosity, strain, or dissipated power. In the filled sections of the extruder, the flow results from the shear due to the rotation of the screws and the local pressure gradient. If one considers only the screw elements of a corotating intermeshing twin-screw extruder (right-handed or left-handed, a right-handed element has a flight angle such as the screw rotation pushes the material downstream. In contrast, a left-handed element has a flight angle such has the screw rotation pushes the material upstream), the flow conditions are very similar to those in the metering zone of a single-screw extruder [3]. In this case, the average shear rate $\bar{\dot{\gamma }}$ is usually expressed as [4,5,6,7,8]:

(1) $$\bar{\dot{\gamma }}=\frac{2\pi N\;R}{60\;h}$$

where *N* is the rotation speed (expressed in rpm), *R* is the screw radius, and *h* is the channel depth.

This expression means that only the shear flow is considered and the pressure flow is neglected. This assumption is often valid in single-screw extrusion, where pressure gradients are usually low. However, this is no longer the case in twin-screw extrusion, especially in left-handed elements, where values of a few hundred MPa/m are common. It is therefore necessary to include a pressure term in the calculation of the average shear rate. To our knowledge, the literature is rather limited on this subject and only a few papers are available. For example, Mohamed et al. [9] proposed the following expression for a corotating twin-screw extruder:

(2) $$\;\bar{\dot{\gamma }}=\beta_{1}\;N^{\beta_{2}}Q^{\beta_{3}}$$

where *Q* is the mass flow rate and *β*_1_, *β*_2_, and *β*_3_ are constants depending on the screw configuration. If the term *Q^β^*^3^ allows to consider the effect of pressure, the constants must be determined experimentally, which limits the usefulness of the formulation. Another expression of the same type, but without pressure term, has been later proposed by Suparno et al. [10]:

(3) $$\;\bar{\dot{\gamma }}=k^{\prime }\;N^{\alpha }$$

where *k*′ is a characteristic of the extruder and α is a parameter that increases with the power-law index and is a function of the filling ratio. Like the previous one, this expression lacks generality and cannot be easily used, without a preliminary experimental determination of the different constants.

Therefore, the objective of the present paper is to propose a general method for defining the average value of the shear rate in the screw elements of a corotating twin-screw extruder, as a function of the screw geometry and the processing parameters controlled by the user, i.e., screw speed and feed rate. Two methods, approximate and exact, are thus developed and compared.

## 2. Assumptions and Description of the Problem

**Figure 1:**
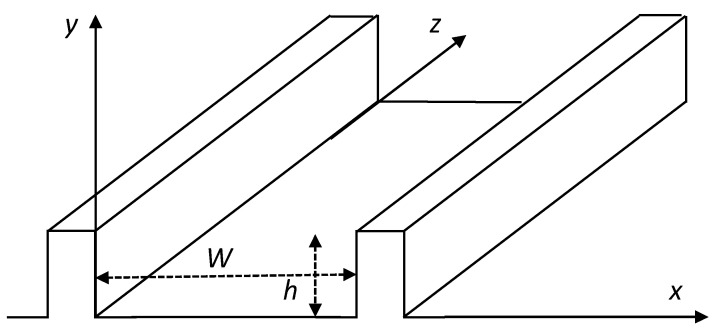
Description of the screw channel and coordinate system.

For the sake of simplicity, we consider the flow of a Newtonian isothermal fluid of viscosity *η* along the screw channel of a twin-screw extruder. The channel along the C-chambers is unrolled and the intermeshing area is neglected, leading to the geometry depicted in Figure 1, which is similar to that of the metering zone of a single screw extruder [1]. The cross section is assumed to be rectangular, with a width *W* and a depth *h*. *W* and *h* can be deduced from the actual geometry of the channel, depending on the screw radius, the centerline distance, the screw pitch and the number of flights, according to the expressions initially proposed by Booy [11].

Depending on the number of flights *n*, 2*n*−1 independent and parallel channels must be considered [3]. In the following, only one channel is analyzed, along which the volumetric flow rate is defined as:

(4) $$Q_{ch}=\frac{Q}{\rho \;(2n-1)}$$

where *Q* is the total mass flow rate and *ρ* is the melt density. The screws are assumed to be fixed and the barrel to move at a velocity *V*, the direction of which makes an angle *θ* with the *z*-axis of the channel. *V* and *θ* are respectively defined as:

(5) $$V=\frac{2\pi N\;R}{60}$$

(6) $$\tan\theta =\frac{B}{2\pi R}$$

where *B* is the screw pitch.

With the classical assumptions of the flow in screw channels [3,8], only two components of the velocity field are considered, which are only a function of the *y*-direction: (*u*(*y*), 0, *w*(*y*)). This makes it possible to decompose the flow into a longitudinal flow along the screw axis and a transversal flow across the screw channel. No-slip on the walls is assumed, which leads to the following boundary conditions:

*u*(0) = 0, *u*(*h*) = −*V* sin*θ, w*(0) = 0, *w*(*h*) = S*V* cos*θ* (7)

*S* is a parameter the value of which is 1 for a right-handed element and −1 for a left-handed element. The integration of the Stokes equations with the boundary conditions (7) leads to the expressions of the velocity components [3]:

(8) $$u(y)=\frac{1}{2\eta }\frac{\partial p}{\partial x}y(y-h)-V\sin\theta \frac{y}{h}$$

(9) $$w(y)=\frac{1}{2\eta }\frac{\partial p}{\partial z}y(y-h)+SV\cos\theta \frac{y}{h}$$

Equation (8) concerns the transverse flow in the *x−y* plane, while Equation (9) concerns the longitudinal flow. Since the transverse flow is a complete recirculation, the transverse flow rate, obtained by integrating Equation (8) between 0 and *h*, is equal to zero. This makes it possible to calculate the transverse pressure gradient *∂p*/*∂x*, and to obtain the final expression of the transverse velocity:

(10) $$u(y)=-V\sin\theta \frac{y}{h}\left(3\frac{y}{h}-2\right)$$

The shear rates in the different shear planes are now deduced by derivation of the velocity components:

(11) $${\dot{\gamma }}_{xy}=\frac{\partial u}{\partial y}=\frac{2V\sin\theta }{h^{2}}\left(h-3y\right)$$

(12) $${\dot{\gamma }}_{yz}=\frac{\partial w}{\partial y}=\frac{1}{2\eta }\frac{\partial p}{\partial z}\left(2y-h\right)+\frac{SV\cos\theta }{h}$$

The objective is now to estimate the average value of these shear rates, especially the shear rate in the flow direction ${\dot{\gamma }}_{yz}$. For this, two methods are proposed.

## 3. Approximate Method

Equation (12) shows that the shear rate ${\dot{\gamma }}_{yz}$ is the sum of a pressure term and a drag term: ${\dot{\gamma }}_{yz}={\dot{\gamma }}_{p}+{\dot{\gamma }}_{d}$, with:

(13) $${\dot{\gamma }}_{p}=\frac{1}{2\eta }\frac{\partial p}{\partial z}\left(2y-h\right)$$

(14) $${\dot{\gamma }}_{d}=\frac{V\cos\theta }{h}$$

To calculate the average value of ${\dot{\gamma }}_{yz}$, which is named ${\bar{\dot{\gamma }}}_{yz}$, it is assumed that ${\bar{\dot{\gamma }}}_{yz}={\bar{\dot{\gamma }}}_{p}+{\bar{\dot{\gamma }}}_{d}$, where ${\bar{\dot{\gamma }}}_{p}$ and ${\bar{\dot{\gamma }}}_{d}$ are the average values of the pressure and shear terms, respectively. This is not rigorously exact, because the average of a sum is not the sum of the averages, but it allows for easier calculations. The average values are obtained by integration over the channel depth. The position where the sign of the shear rate changes (i.e., the location of the maximum velocity) has to be taken into account.

### 3.1. Right-Handed Elements

A right-handed element is usually only partially filled. In this case, ∂p/∂z = 0 and ${\bar{\dot{\gamma }}}_{yz}={\bar{\dot{\gamma }}}_{d}$. The pressure term must be incorporated when the element is fully filled, for example in front of a restrictive element (left-handed element or block of kneading discs) or before the die.

The average values of ${\dot{\gamma }}_{p}$ and ${\dot{\gamma }}_{d}$ are:

(15) $${\bar{\dot{\gamma }}}_{p}=\frac{1}{h}\left[\int_{0}^{h/2}\frac{1}{2\eta }\left|\frac{\partial p}{\partial z}\right|\left(h-2y\right)dy+\int_{h/2}^{h}\frac{1}{2\eta }\left|\frac{\partial p}{\partial z}\right|\left(2y-h\right)dy\right]=\frac{h}{4\eta }\left|\frac{\partial p}{\partial z}\right|$$

(16) $${\bar{\dot{\gamma }}}_{d}=\frac{1}{h}\int_{0}^{h}\frac{V\cos\theta }{h}dy=\frac{V\;\cos\theta }{h}$$

The average shear rate is thus:

(17) $${\bar{\dot{\gamma }}}_{yz}=\frac{h}{4\eta }\left|\frac{\partial p}{\partial z}\right|+\frac{V\;\cos\theta }{h}$$

On a practical point of view, it is the flow rate that is imposed by the user, not the pressure gradient. The relationship between the flow rate and the pressure gradient can be expressed as [12]:

(18) $$\frac{\partial p}{\partial z}=\frac{12\eta }{h^{3}}\left(V\;\cos\theta \frac{h}{2}-\frac{Q_{ch}}{W}\right)$$

where *W* is the channel width. The sign of ∂p/∂z depends on the value of *Q_ch_* in relation to the maximum conveying flow rate Q*, defined as:

(19) $$Q*=V\;\cos\theta \;\frac{h\;W}{2}$$

Therefore, the average shear rate can finally be expressed as:

(20) $${\bar{\dot{\gamma }}}_{yz}=\frac{5}{2}\frac{V\;\cos\theta }{h}-\frac{3Q_{ch}}{Wh^{2}},\;\;if\;Q_{ch}<Q*$$

(21) $${\bar{\dot{\gamma }}}_{yz}=-\frac{1}{2}\frac{V\;\cos\theta }{h}+\frac{3Q_{ch}}{Wh^{2}},\;\;if\;Q_{ch}>Q*$$

**Figure 2:**
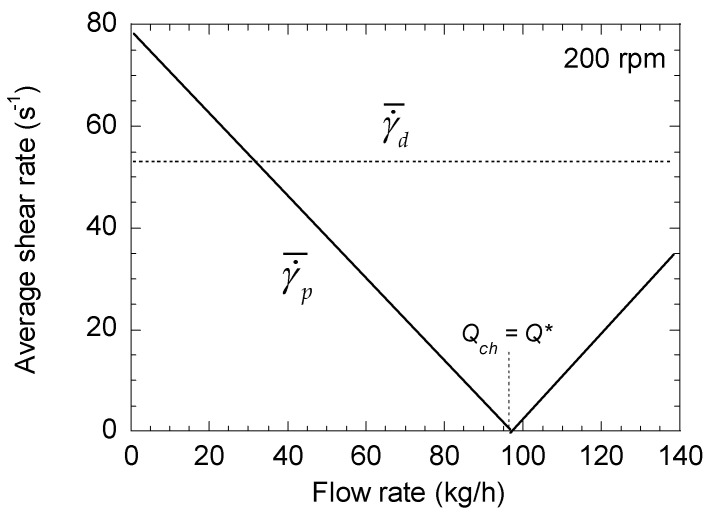
Changes in the average shear rates (pressure and drag terms) of a right-handed element as a function of total flow rate for a screw speed of 200 rpm.

**Figure 3:**
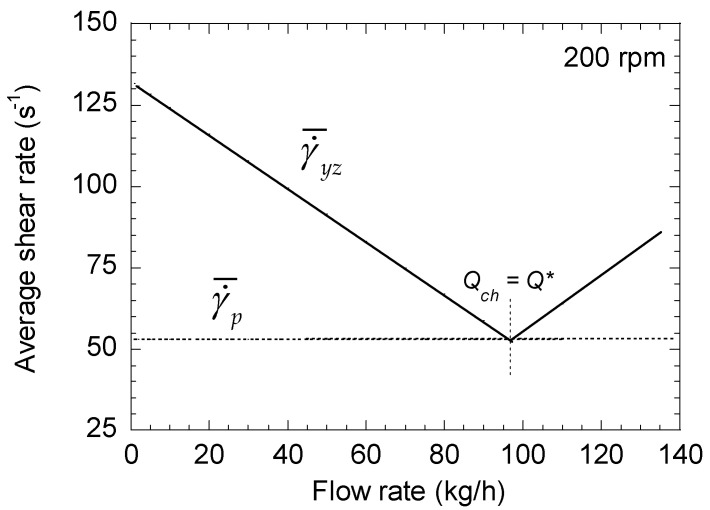
Changes in the average shear rates of a right-handed element as a function of total flow rate for a screw speed of 200 rpm. Comparison between total shear rate and drag term.

To illustrate these results, let us consider the case of a lab scale twin-screw extruder Leistritz ZSE 27 MAXX, with a screw radius of 14.15 mm and a screw pitch of 30 mm. The channel dimensions are h = 5.3 mm and W = 12.7 mm. The extruded material has a viscosity of 1000 Pa·s and a density of 950 kg/m^3^. Figure 2 shows the changes in ${\bar{\dot{\gamma }}}_{p}$ and ${\bar{\dot{\gamma }}}_{d}$ with the flow rate for a screw speed of 200 rpm. While ${\bar{\dot{\gamma }}}_{d}$ is constant, ${\bar{\dot{\gamma }}}_{p}$ decreases linearly with the flow rate until *Q* = Q*, where its value is null. Above this point, ${\bar{\dot{\gamma }}}_{p}$ increases with *Q*, with the same slope. Similar results are obtained for the other screw speeds. Significant differences between the two terms ${\bar{\dot{\gamma }}}_{p}$ and ${\bar{\dot{\gamma }}}_{d}$ are observed, indicating the need to consider the pressure term for a correct estimation of the average shear rate. The comparison between ${\bar{\dot{\gamma }}}_{yz}$ and ${\bar{\dot{\gamma }}}_{d}$ in Figure 3 confirms that a factor higher than 2 may exist between the two values, regardless of the screw speed, and that neglecting the pressure term leads to a strong underestimation of the average shear rate.

### 3.2. Left-Handed Elements

Left-handed elements are always filled and under pressure, and the pressure gradient is always negative, whatever the value of *Q_ch_*:

(22) $$\frac{\partial p}{\partial z}=\frac{12\eta }{h^{3}}\left(-V\;\cos\theta \frac{h}{2}-\frac{Q_{ch}}{W}\right)$$

Therefore, according to Equation (17):

(23) $${\bar{\dot{\gamma }}}_{yz}=\frac{5}{2}\frac{V\;\cos\theta }{h}+\frac{3Q_{ch}}{Wh^{2}}$$

**Figure 4:**
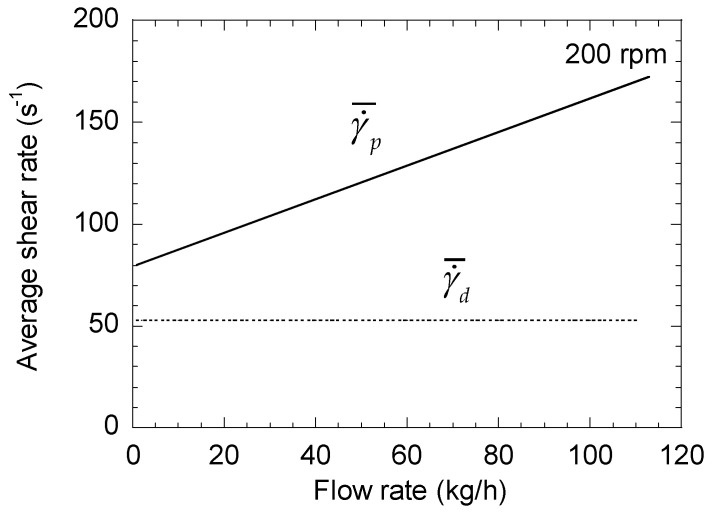
Changes in the average shear rates (pressure and drag terms) of a left-handed element as a function of total flow rate for a screw speeds of 200 rpm.

**Figure 5:**
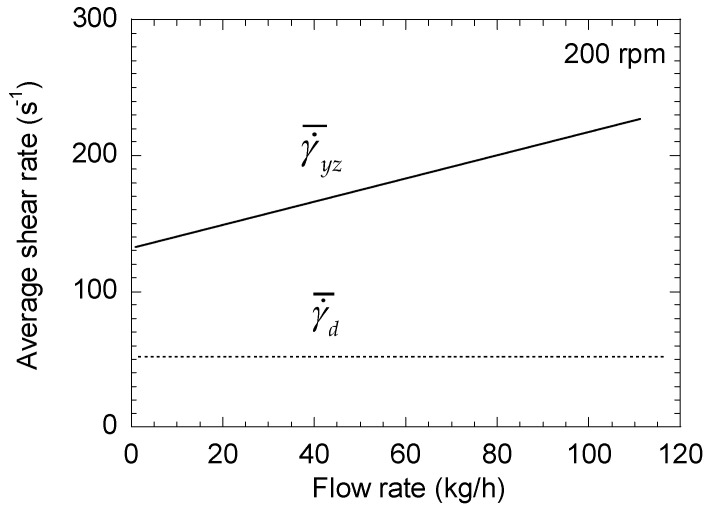
Changes in the average shear rates of a left-handed element as a function of total flow rate for a screw speeds of 200 rpm. Comparison between total shear rate and drag term.

In this case, using the same data as for the right-handed elements, it can be seen in Figure 4 that ${\bar{\dot{\gamma }}}_{p}$ increases continuously with the flow rate. As a consequence, the difference between ${\bar{\dot{\gamma }}}_{yz}$ and ${\bar{\dot{\gamma }}}_{d}$ is much larger and can reach a factor of 3 to 4 (Figure 5).

## 4. Exact Method

To calculate the average shear rate exactly, Equation (12) must be integrated:

(24) $${\bar{\dot{\gamma }}}_{yz}=\frac{1}{h}\int_{0}^{h}\left|{\dot{\gamma }}_{yz}\right|dy=\frac{1}{h}\int_{0}^{h}\left|\frac{1}{2\eta }\frac{\partial p}{\partial z}\left(2y-h\right)+\frac{SV\;\cos\theta }{h}\right|dy$$

Here, the main difficulty is to define the sign of ${\dot{\gamma }}_{yz}$ over the interval [0, *h*].

### 4.1. Right-Handed Elements

For a fully filled right-handed element, the sign of ${\dot{\gamma }}_{yz}$ changes for a value h* equal to:

(25) $$h*=\frac{h}{2}-\frac{V\;\eta \;\cos\theta }{h\;\partial p/\partial z}$$

Two cases have to be considered depending on the sign of $\frac{\partial p}{\partial z}$.

#### 4.1.1. Positive Pressure Gradient: $\frac{\partial p}{\partial z}$ > 0

In this case, h* is less than *h*/2. h* is positive if $\frac{\partial p}{\partial z}\geq \frac{2\;V\;\eta \cos\theta }{h{\;}^{2}}$. Then, ${\dot{\gamma }}_{yz}$ is negative between 0 and h*, and positive between h* and *h*. Therefore:

(26) $${\bar{\dot{\gamma }}}_{yz}=\frac{1}{h}\left[\int_{0}^{h*}\left(-\frac{1}{2\eta }\frac{\partial p}{\partial z}\left(2y-h\right)-\frac{V\;\cos\theta }{h}\right)dy+\int_{h*}^{h}\left(\frac{1}{2\eta }\frac{\partial p}{\partial z}\left(2y-h\right)+\frac{V\;\cos\theta }{h}\right)dy\right],$$

which gives:

(27) $${\bar{\dot{\gamma }}}_{yz}=\frac{h}{4\eta }\frac{\partial p}{\partial z}+\frac{\eta \;V^{2}\;\cos^{2}\theta }{h^{3}\frac{\partial p}{\partial z}}.$$

If $\frac{\partial p}{\partial z}\leq \frac{2\;V\;\eta \cos\theta }{h{\;}^{2}}$, h* is negative and ${\dot{\gamma }}_{yz}$ is positive over the whole interval. Then:

(28) $${\bar{\dot{\gamma }}}_{yz}=\frac{1}{h}\int_{0}^{h}\left(\frac{1}{2\eta }\frac{\partial p}{\partial z}\left(2y-h\right)+\frac{V\;\cos\theta }{h}\right)dy=\frac{V\;\cos\theta }{h}$$

It is the expression for a simple drag flow, indicating that, in this case, the pressure does not play a role; it increases the shear rate on one part of the flow and decreases it on the other part, and, finally, the two effects perfectly compensate.

#### 4.1.2. Negative Pressure Gradient: $\frac{\partial p}{\partial z}$ < 0

According to Equation (25), h* is greater than *h*/2. To be less than *h*, it is necessary that $\left(-\frac{\partial p}{\partial z}\right)\geq \frac{2\;V\;\eta \cos\theta }{h{\;}^{2}}$. ${\dot{\gamma }}_{yz}$ is then positive between 0 and h*, and negative between h* and *h*. Therefore:

(29) $${\bar{\dot{\gamma }}}_{yz}=\frac{1}{h}\left[\int_{0}^{h*}\left(\frac{1}{2\eta }\frac{\partial p}{\partial z}\left(2y-h\right)+\frac{V\;\cos\theta }{h}\right)dy+\int_{h*}^{h}\left(-\frac{1}{2\eta }\frac{\partial p}{\partial z}\left(2y-h\right)-\frac{V\;\cos\theta }{h}\right)dy\right],$$

and finally:

(30) $${\bar{\dot{\gamma }}}_{yz}=\frac{h}{4\eta }\left(-\frac{\partial p}{\partial z}\right)+\frac{\eta \;V^{2}\;\cos^{2}\theta }{h^{3}\left(-\frac{\partial p}{\partial z}\right)}$$

If $\left(-\frac{\partial p}{\partial z}\right)\leq \frac{2\;V\;\eta \cos\theta }{h{\;}^{2}}$, h* is greater than *h* and ${\dot{\gamma }}_{yz}$ is positive over the whole interval. This is the same case as for Equation (28), and the result is obviously the same.

### 4.2. Left-Handed Elements

The shear rate in a left-handed element is given by Equation (12), with *S* = −1:

(31) $${\dot{\gamma }}_{yz}=\frac{1}{2\eta }\frac{\partial p}{\partial z}\left(2y-h\right)-\frac{V\;\cos\theta }{h}$$

The value of h* is thus now:

(32) $$h*=\frac{h}{2}+\frac{V\;\eta \;\cos\theta }{h\;\partial p/\partial z}$$

As ∂p/∂z is always negative, h* is less than *h*/2, and two cases must be considered.

#### 4.2.1. High Pressure Gradient: $\left(-\frac{\partial p}{\partial z}\right)\geq \frac{2V\;\eta \;\cos\theta }{h^{2}}$

Here, h* is positive. ${\dot{\gamma }}_{yz}$ is then positive between 0 and h*, and negative between h* and *h*. Therefore:

(33) $${\bar{\dot{\gamma }}}_{yz}=\frac{1}{h}\left[\int_{0}^{h*}\left(\frac{1}{2\eta }\frac{\partial p}{\partial z}\left(2y-h\right)-\frac{V\;\cos\theta }{h}\right)dy+\int_{h*}^{h}\left(-\frac{1}{2\eta }\frac{\partial p}{\partial z}\left(2y-h\right)+\frac{V\;\cos\theta }{h}\right)dy\right],$$

which leads to:

(34) $${\bar{\dot{\gamma }}}_{yz}=\frac{h}{4\eta }\left(-\frac{\partial p}{\partial z}\right)+\frac{\eta \;V^{2}\;\cos^{2}\theta }{h^{3}\left(-\frac{\partial p}{\partial z}\right)}$$

#### 4.2.2. Low Pressure Gradient: $\left(-\frac{\partial p}{\partial z}\right)\leq \frac{2V\;\eta \cos\theta }{h^{2}}$

h* is now negative and ${\dot{\gamma }}_{yz}$ is negative between 0 and *h*. Consequently:

(35) $${\bar{\dot{\gamma }}}_{yz}=\frac{1}{h}\int_{0}^{h}\left(-\frac{1}{2\eta }\frac{\partial p}{\partial z}\left(2y-h\right)+\frac{V\;\cos\theta }{h}\right)dy=\frac{V\;\cos\theta }{h}$$

We find again the expression for a simple drag flow. However, if we consider the expression of ∂p/∂z (Equation (22)), it can be shown that this condition can never been met for a left-handed element. Therefore, this case can be forgotten.

### 4.3. Summary

Despite the various possible configurations, only two expressions are obtained for the average shear rate, depending on the relative values of the pressure gradient and the screw speed:

(36) $$If\;\left|\frac{\partial p}{\partial z}\right|\leq \frac{2\;V\;\eta \cos\theta }{h^{2}},{\bar{\dot{\gamma }}}_{yz}=\frac{V\;\cos\theta }{h},$$

(37) $$If\;\left|\frac{\partial p}{\partial z}\right|\geq \frac{2\;V\;\eta \cos\theta }{h^{2}},{\bar{\dot{\gamma }}}_{yz}=\frac{h}{4\eta }\left|\frac{\partial p}{\partial z}\right|+\frac{\eta \;V^{2}\;\cos^{2}\theta }{h^{3}\left|\frac{\partial p}{\partial z}\right|}$$

The pressure gradient can be expressed as function of screw speed and flow rate *Q_ch_* by Equations (18) and (22). By using these expressions, it is possible to summarize the different cases according to the relationships between *Q_ch_* and the maximum conveying flow rate Q* (Equation (19)). The results are presented in Table 1. Finally, a single expression is obtained for the left-handed elements, while two are required for the right-handed ones.

For $Q_{ch}\leq \frac{2}{3}Q*$ and $Q_{ch}\geq \frac{4}{3}Q*$, ${\dot{\gamma }}_{yz}$ has the same expression for the right- and left-handed elements. However, for the same flow rate, the pressure gradient is higher for a left-handed element (compare Equations (18) and (22)). Therefore, under the same processing conditions, a left-handed element will always provide a higher shear rate than a right-handed element of the same geometry. This effect would not be accounted for by using only a drag term in the average value of the shear rate.

**Figure 6:**
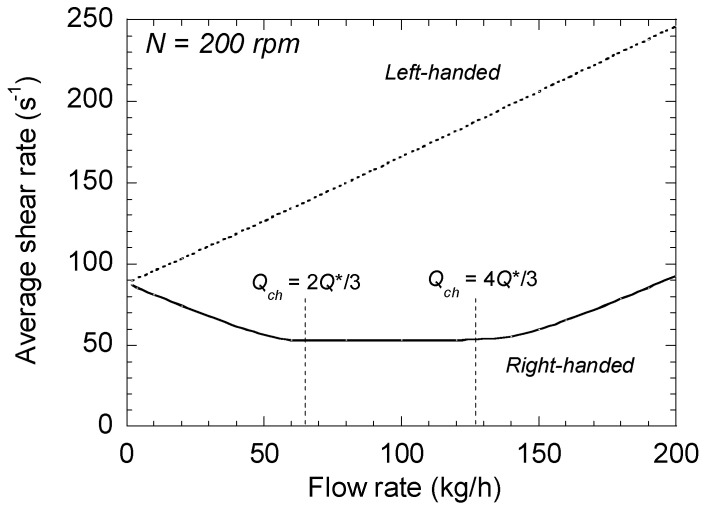
Changes in the average shear rates of right-and left-handed elements as a function of total flow rate at 200 rpm.

To illustrate these results, the same application as in the previous section is used. Figure 6 shows the variations of average shear rate with the flow rate, for right- and left-handed elements of the same geometry at 200 rpm. For the right-handed screw element, ${\bar{\dot{\gamma }}}_{yz}$ first decreases with the flow rate up to 2Q*/3. Then, between 2Q*/3 and 4Q*/3, the shear rate is constant and equal to the value of the drag term. Above 4Q*/3, ${\bar{\dot{\gamma }}}_{yz}$ increases linearly with the flow rate. In contrast, for the left-handed element, the average shear rate increases continuously, and, as explained above, is always higher than that of the right-handed element.

**Figure 7:**
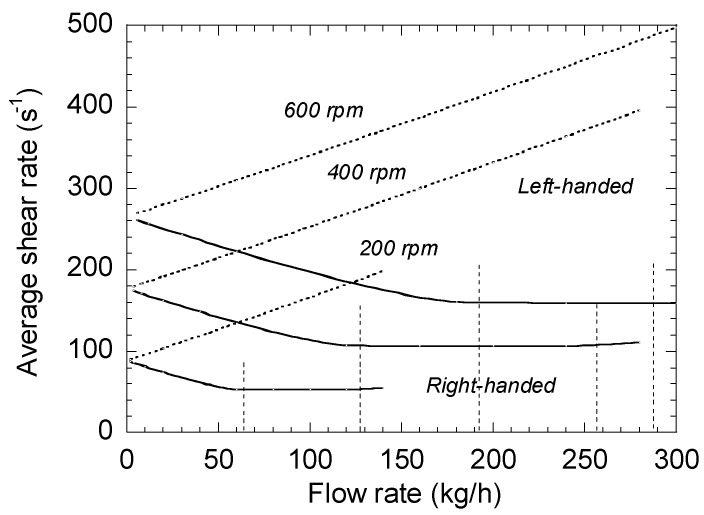
Influence of the screw speed on the average shear rate of right- and left-handed elements.

The influence of the screw speed *N* is shown in Figure 7. When *N* increases, the values of ${\bar{\dot{\gamma }}}_{yz}$ increase, but the overall trends remain the same. The limits of the zone where the shear rate is constant also increase with the screw speed.

## 5. Comparison between the Two Methods and Complements

### 5.1. Comparison between the Two Methods

**Figure 8:**
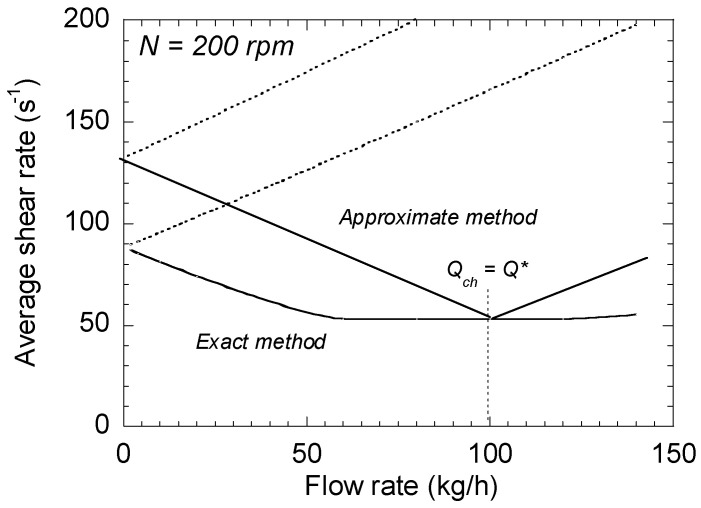
Comparison between the results provided by the two methods at 200 rpm.

A comparison between exact and approximate methods is proposed in Figure 8 for a screw speed of 200 rpm. It must be noted that the results are similar regardless of the screw speed. First of all, the orders of magnitude provided by the two methods are in agreement. However, some differences are put in evidence. A shear rate plateau of minimal value exists for the exact method, while only a minimum appears for the approximated one, for *Q_ch_* = Q*. In both cases, the minimum corresponds to the simple drag shear, which is the assumption generally admitted in the literature for this type of flow.

For both right- and left-handed elements, the results of the approximate method always overestimate that of the exact one, by up to 50% at low flow rate. They are nevertheless more accurate than those obtained by using the assumption of simple drag flow.

### 5.2. Calculation of the Total Shear Rate

So far, only the longitudinal flow along the screw channel has been considered. To correctly estimate the average shear rate, the transverse flow in the *x−y* plane must also be taken into account. The transverse shear rate is defined by Equation (38):

(38) $${\dot{\gamma }}_{xy}=\frac{2V\;\sin\theta }{h^{2}}\left(h-3y\right)$$

Here, the situation is simple: h* = *h*/3 and ${\dot{\gamma }}_{xy}$ is first positive between 0 and h*, then negative between h* and *h*. Therefore:

(39) $${\bar{\dot{\gamma }}}_{xy}=\frac{1}{h}\left[\int_{0}^{h/3}{\dot{\gamma }}_{xy}\;dy+\int_{h/3}^{h}(-{\dot{\gamma }}_{xy})\;dy\right]=\frac{5}{3}\frac{V\;\sin\theta }{h}$$

This expression is valid whatever the configuration, i.e., for right-and left-handed elements. The global average shear rate is thus given by:

(40) $$\bar{\dot{\gamma }}=\sqrt{{\bar{\dot{\gamma }}}_{xy}{}^{2}+{\bar{\dot{\gamma }}}_{yz}{}^{2}},$$

where ${\bar{\dot{\gamma }}}_{xy}$ is given by Equation (39) and ${\bar{\dot{\gamma }}}_{yz}$ by Equations (36) or (37).

Generally speaking, ${\bar{\dot{\gamma }}}_{xy}$ is always much lower than ${\bar{\dot{\gamma }}}_{yz}$. According to Equation (39), ${\bar{\dot{\gamma }}}_{xy}$ value is only 56% of the minimum value of ${\bar{\dot{\gamma }}}_{yz}$, reached on the plateau for a right-handed element. For a left-handed element, ${\bar{\dot{\gamma }}}_{yz}$ is at least three times higher than ${\bar{\dot{\gamma }}}_{xy}$, and the difference increases with the flow rate. Therefore, the transverse shear rate could be neglected in a first approximation, the maximum error being about 10%.

## 6. Conclusions

In this paper, the average shear rate in the screw channel of a corotating twin-screw extruder was evaluated. Two methods, approximate and exact, were proposed. The comparison shows that they are in agreement, even though the exact method provides more accurate results. The effects of screw speed and flow rate were investigated. In all cases, under identical processing conditions, the shear rates of the left-handed elements are always higher than those of the right-handed elements. The usual approximation of the shear rate by considering only a simple drag flow, neglecting the pressure term, proves to be incorrect, leading to a strong underestimation and a lack of distinction between right- and left-handed elements.

## Figures and Tables

**Table 1:** Values of average shear rates for right- and left-handed elements.

	Left-Handed Elements		Right-Handed Elements	
Flow rate	Pressure gradient ∂p/∂z	Shear rate ${\dot{\gamma }}_{yz}$	Pressure gradient ∂p/∂z	Shear rate ${\dot{\gamma }}_{yz}$
$Q_{ch}\leq \frac{2}{3}Q*$	<0	$\frac{h}{4\eta }\left|\frac{\partial p}{\partial z}\right|+\frac{\eta \;V^{2}\;\cos^{2}\theta }{h^{3}\left|\frac{\partial p}{\partial z}\right|}$	>0	$\frac{h}{4\eta }\left|\frac{\partial p}{\partial z}\right|+\frac{\eta \;V^{2}\;\cos^{2}\theta }{h^{3}\left|\frac{\partial p}{\partial z}\right|}$
$\frac{2}{3}Q*\leq Q_{ch}\leq Q*$	<0	$\frac{h}{4\eta }\left|\frac{\partial p}{\partial z}\right|+\frac{\eta \;V^{2}\;\cos^{2}\theta }{h^{3}\left|\frac{\partial p}{\partial z}\right|}$	>0	$\frac{V\;\cos\theta }{h}$
$Q*\leq Q_{ch}\leq \frac{4}{3}Q*$	<0	$\frac{h}{4\eta }\left|\frac{\partial p}{\partial z}\right|+\frac{\eta \;V^{2}\;\cos^{2}\theta }{h^{3}\left|\frac{\partial p}{\partial z}\right|}$	<0	$\frac{V\;\cos\theta }{h}$
$\frac{4}{3}Q*\leq Q_{ch}$	<0	$\frac{h}{4\eta }\left|\frac{\partial p}{\partial z}\right|+\frac{\eta \;V^{2}\;\cos^{2}\theta }{h^{3}\left|\frac{\partial p}{\partial z}\right|}$	<0	$\frac{h}{4\eta }\left|\frac{\partial p}{\partial z}\right|+\frac{\eta \;V^{2}\;\cos^{2}\theta }{h^{3}\left|\frac{\partial p}{\partial z}\right|}$

## Data Availability

Not applicable.
